# Detecting Mpox Cases Through Wastewater Surveillance — United States, August 2022–May 2023

**DOI:** 10.15585/mmwr.mm7302a3

**Published:** 2024-01-18

**Authors:** Carly Adams, Amy E. Kirby, Megan Bias, Aspen Riser, Karen K. Wong, Jeffrey W. Mercante, Heather Reese

**Affiliations:** ^1^Division of Infectious Disease Readiness and Innovation, National Center for Emerging and Zoonotic Infectious Diseases, CDC; ^2^Epidemic Intelligence Service, CDC; ^3^Division of HIV Prevention, National Center for HIV, Viral Hepatitis, STD, and TB Prevention, CDC; ^4^National Center for Emerging and Zoonotic Infectious Diseases, Office of the Director, CDC.

**Summary**

**What is already known about this topic?**

CDC’s National Wastewater Surveillance System began testing wastewater for *Monkeypox* *virus* in October 2022. The performance of wastewater surveillance for detecting mpox cases is unknown.

**What is added by this report?**

*Monkeypox* *virus* wastewater detections were compared with reported mpox cases. Wastewater surveillance has a sensitivity of 32% for detecting a single mpox case in wastewater samples that represent thousands to millions of persons. Sensitivity increases as the number of cases in the community increases. Positive and negative predictive values are high.

**What are the implications for public health practice?**

An isolated *Monkeypox* *virus* wastewater detection likely warrants a limited public health response. Absence of *Monkeypox* *virus* detection in a monitored community can provide reassurance that large numbers of cases are not present. *Monkeypox* *virus* wastewater surveillance is a useful complement to mpox case surveillance.

## Abstract

In October 2022, CDC’s National Wastewater Surveillance System began routine testing of U.S. wastewater for *Monkeypox* *virus*. Wastewater surveillance sensitivity, positive predictive value (PPV), and negative predictive value (NPV) for *Monkeypox* *virus* were evaluated by comparing wastewater detections (*Monkeypox* *virus* detected versus not detected) to numbers of persons with mpox in a county who were shedding virus. Case ascertainment was assumed to be complete, and persons with mpox were assumed to shed virus for 25 days after symptom onset. A total of 281 cases and 3,492 wastewater samples from 89 sites in 26 counties were included in the analysis. Wastewater surveillance in a single week, from samples representing thousands to millions of persons, had a sensitivity of 32% for detecting one or more persons shedding *Monkeypox* *virus*, 49% for detecting five or more persons shedding virus, and 77% for detecting 15 or more persons shedding virus. Weekly PPV and NPV for detecting persons shedding *Monkeypox* *virus* in a county were 62% and 80%, respectively. An absence of detections in counties with wastewater surveillance signified a high probability that a large number of cases were not present. Results can help to guide the public health response to *Monkeypox* *virus* wastewater detections. A single, isolated detection likely warrants a limited public health response. An absence of detections, in combination with no reported cases, can give public health officials greater confidence that no cases are present. Wastewater surveillance can serve as a useful complement to case surveillance for guiding the public health response to an mpox outbreak.

## Introduction

The global mpox outbreak began in May 2022 when mpox began spreading widely outside countries with endemic transmission.* Persons with mpox can shed *Monkeypox* *virus* DNA in skin lesions, urine, and stool; thus, *Monkeypox* *virus* infections can be tracked through wastewater surveillance (*1*). In October 2022, CDC’s National Wastewater Surveillance System (NWSS),^†^ which was established during the COVID-19 pandemic, began testing U.S. wastewater for *Monkeypox virus*. By May 2023, more than 500 sampling sites in 49 states were testing wastewater for *Monkeypox* *virus*.^§^ The goal of this analysis was to evaluate the sensitivity, positive predictive value (PPV), and negative predictive value (NPV) of wastewater surveillance for detecting mpox cases in the United States.

## Methods

### Data Sources

Sample-level NWSS wastewater data from August 2022–May 2023 (as of June 2023) were analyzed. *Monkeypox* *virus* wastewater data were reported to NWSS by a commercial contractor and an academic partner program; these entities began *Monkeypox* *virus* testing in October 2022 and June 2022, respectively^¶^ (*2**,**3*). Wastewater surveillance data were compared with case surveillance data** from the same period, as of July 2023. CDC mpox case definitions were used,^††^ and both confirmed and probable cases were included.

### Data Analysis

Because of differences in reporting units for wastewater surveillance and case data, analyses were conducted by county and were restricted to counties with ≥90% population coverage by wastewater surveillance (Supplementary Figure 1, https://stacks.cdc.gov/view/cdc/140513). Population coverage was determined by dividing estimated numbers of persons served by all sampling sites in a county by the county population^§§^ (*4**,**5*). Only dates during which all sites serving a county were collecting samples were included in the analysis. Exact dates of inclusion varied by county.^¶¶^ Overall, included samples were collected during August 2022–May 2023.

Persons with mpox were assumed to shed virus uniformly for 25 days from the date of symptom onset*** (*1*). Missing symptom onset dates (for approximately 20% of cases) were imputed by subtracting empirical median lag times (time between symptom onset and other clinical dates^†††^) from the earliest date available for each case (Supplementary Table, https://stacks.cdc.gov/view/cdc/140513).^§§§^ Mpox case ascertainment was assumed to be complete, and only persons with confirmed or probable mpox were assumed to be shedding virus (*6*). Dichotomous county wastewater results (*Monkeypox* *virus* detection versus nondetection) were compared with the number of persons presumed to be shedding virus in the county on the sample collection date. If *Monkeypox* *virus* was detected in at least one sample from a county on a given day or week, that day or week was considered a virus detection day or week; if no *Monkeypox* *virus* was detected, that day or week was classified as a nondetection.

Sensitivity, PPV, and NPV of wastewater surveillance on a single day or week for detecting persons shedding *Monkeypox* *virus* in a county were calculated.^¶¶¶^ Sensitivity was defined as the probability of a wastewater detection assuming that one or more persons was shedding virus. Sensitivities for detecting varying minimum numbers of persons shedding virus were also calculated. PPV was defined as the probability that at least one person was shedding virus when a wastewater detection occurred, and NPV was defined as the probability that no persons were shedding virus in the absence of wastewater detections. The probabilities that different numbers of persons were shedding virus given the presence or absence of wastewater detections were also examined.****

Three sensitivity analyses were performed. First, the assumed shedding duration was varied from 5 to 60 days in 5-day increments. Second, the earliest date available for all cases was used, rather than imputed symptom onset dates. Third, rolling 7-day average estimates were calculated,^††††^ rather than weekly estimates. Analyses were conducted using R software (version 4.2.3; R Foundation). This activity was reviewed by CDC, deemed not research, and was conducted consistent with applicable federal law and CDC policy.^§§§§^

## Results

A total of 3,492 wastewater samples from 89 sites and 26 counties (16 states) were included in the analysis (Table 1). *Monkeypox* *virus* DNA was detected in 95 samples (3%) from 17 counties (65%); 281 cases from 12 counties were included.^¶¶¶¶^

**TABLE 1:** Information on population, *Monkeypox virus* wastewater samples, and persons with mpox during the study period for counties included in the analysis* — United States, August 2022–May 2023

County^†^	Population^§^	No. of wastewater samples	Average no. of samples collected per site per week (range)	No. of days with MPXV wastewater detections	No. of persons with mpox included^¶^	Wastewater sample collection date range**
1	250,000–999,999	487	2.9 (1–4)	29	70–79	Aug 2022–May 2023
2	250,000–999,999	73	1.4 (1–2)	6	20–29	Oct 2022–May 2023
3	≥1,000,000	215	1.7 (1–2)	6	60–69	Oct 2022–May 2023
4	≥1,000,000	172	1.7 (1–2)	9	50–59	Oct 2022–May 2023
5	20,000–249,999	20	1.1 (1–2)	7	0	Oct 2022–Mar 2023
6	2,500–19,999	104	1.4 (1–2)	0	0	Oct 2022–May 2023
7	250,000–999,999	80	1.8 (1–2)	4	1–9	Nov 2022–May 2023
8	250,000–999,999	194	1.8 (1–3)	0	0	Nov 2022–May 2023
9	20,000–249,999	107	1.7 (1–3)	1	0	Nov 2022–May 2023
10	20,000–249,999	31	1.4 (1–2)	0	0	Nov 2022–May 2023
11	20,000–249,999	33	1.6 (1–2)	3	1–9	Nov 2022–May 2023
12	20,000–249,999	38	1.7 (1–2)	0	0	Nov 2022–May 2023
13	20,000–249,999	34	1.5 (1–2)	0	0	Nov 2022–May 2023
14	250,000–999,999	123	1.6 (1–3)	1	1–9	Nov 2022–May 2023
15	250,000–999,999	360	2.4 (1–4)	1	0	Dec 2022–May 2023
16	250,000–999,999	322	2.0 (1–3)	1	0	Dec 2022–May 2023
17	≥1,000,000	77	2.8 (1–3)	1	20–29	Dec 2022–Mar 2023
18	250,000–999,999	160	1.6 (1–3)	6	1–9	Dec 2022–May 2023
19	≥1,000,000	229	2.4 (1–4)	3	10–19	Jan–May 2023
20	≥1,000,000	77	7.0 (7–7)	1	0	Feb–May 2023
21	≥1,000,000	306	7.0 (6–7)	0	1–9	Feb–May 2023
22	20,000–249,999	57	2.8 (2–3)	0	0	Feb–May 2023
23	≥1,000,000	106	1.5 (1–3)	4	10–19	Mar–May 2023
24	≥1,000,000	69	2.1 (1–3)	1	0	Mar–May 2023
25	20,000–249,999	12	3.0 (3–3)	0	0	Apr–May 2023
26	20,000–249,999	6	2.0 (2–2)	0	0	Apr–May 2023
**Total**	**24,700,152**	**3,492**	**2.2 (1–7)**	**84**	**281**	**Aug 2022–May 2023**

**Abbreviation**: MPXV = *Monkeypox virus*.

* Twenty-six counties that had ≥90% population coverage by wastewater sampling sites testing for MPXV were included in the analysis.

^†^ County numbers were arbitrarily assigned.

^§^ County population estimates were obtained from 2022 U.S. Census Bureau population estimates or 2021 state health department population estimates where U.S. Census Bureau estimates by county were not available.

^¶^ Numbers of persons with mpox included in the main analysis (assuming persons with mpox shed virus from the date of symptom onset until 25 days later) are shown. This number includes persons with mpox with symptom onset dates (reported or imputed) within 25 days before the first study inclusion date through the last study inclusion date for the county of residence.

** Date ranges include dates during which all sites in a county were collecting wastewater samples for MPXV testing. Samples from a county were included in the analysis if they were collected within the county’s data range. The earliest inclusion date per county was the maximum of the minimum sample collection dates for all sites in the county (minus 6 days to account for sampling on different days of the week). The last inclusion date per county was the minimum of the maximum sample collection dates for all sites in the county (plus 6 days), or May 7, 2023, (1 month before data download), whichever date was earliest. For duplicate sites (sites collecting samples for two different sources), dates were eligible for inclusion if at least one source was collecting samples on that date.

### Sensitivity

Sensitivity of wastewater surveillance increased as the number of persons shedding *Monkeypox* *virus* increased. The probability that *Monkeypox* *virus* was detected in wastewater on a given day was 13.8% (95% CI = 10.7%–17.4%) when at least one person was shedding virus, 28.9% (95% CI = 21.9%–36.8%) when five or more persons were shedding virus, and 48.3% (95% CI = 35.2%–61.6%) when 15 or more persons were shedding virus (Table 2). When examining sensitivity during a given week, these estimates increased to 31.7% (95% CI = 23.6%–40.7%), 48.9% (95% CI = 33.7%–64.2%), and 76.5% (95% CI = 50.1%–93.2%), respectively.

**TABLE 2:** Sensitivity, positive predictive value, and negative predictive value* of wastewater surveillance^†^ for detecting persons shedding *Monkeypox virus* in a county, by day and week — United States, August 2022–May 2023

No. of persons shedding MPXV^§^	Daily estimates,^¶^ % (95% CI)		Weekly estimates,** % (95% CI)		
	Main analysis^††^	Analysis using earliest date^§§^	Main analysis^††^	Analysis using earliest date^§§^	Analysis of 7-day rolling average^¶¶^
**Sensitivity (probability of MPXV detection in wastewater when no. of persons with mpox were shedding MPXV)**					
≥1	13.8 (10.7–17.4)	13.7 (10.7–17.2)	31.7 (23.6–40.7)	31.0 (23.0–39.8)	29.2 (24.8–33.9)
≥5	28.9 (21.9–36.8)	29.1 (22.0–37.1)	48.9 (33.7–64.2)	53.3 (37.9–68.3)	54.1 (45.7–62.4)
≥10	37.9 (27.7–49.0)	37.5 (27.4–48.5)	65.2 (42.7–83.6)	64.0 (42.5–82.0)	69.9 (58.8–79.5)
≥15	48.3 (35.2–61.6)	46.9 (34.3–59.8)	76.5 (50.1–93.2)	77.8 (52.4–93.6)	81.7 (69.6–90.5)
≥20	60.0 (44.3–74.3)	57.8 (42.2–72.3)	—	—	—
≥25	63.0 (42.4–80.6)	61.5 (40.6–79.8)	—	—	—
≥30	58.3 (27.7–84.8)	63.6 (30.8–89.1)	—	—	—
**PPV (probability of no. of persons with mpox shedding MPXV when MPXV was detected in wastewater)**					
≥1	72.6 (61.8–81.8)	73.8 (63.1–82.8)	61.9 (48.8–73.9)	61.9 (48.8–73.9)	65.9 (58.6–72.8)
≥5	52.4 (41.2–63.4)	52.4 (41.2–63.4)	34.9 (23.3–48.0)	38.1 (26.1–51.2)	43.4 (36.1–50.9)
≥10	39.3 (28.8–50.5)	39.3 (28.8–50.5)	23.8 (14.0–36.2)	25.4 (15.3–37.9)	31.9 (25.2–39.2)
**≥**15	34.5 (24.5–45.7)	35.7 (25.6–46.9)	20.6 (11.5–32.7)	22.2 (12.7–34.5)	26.9 (20.6–34.0)
≥20	32.1 (22.4–43.2)	31.0 (21.3–42.0)	—	—	—
≥25	20.2 (12.3–30.4)	19.0 (11.3–29.1)	—	—	—
≥30	8.3 (3.4–16.4)	8.3 (3.4–16.4)	—	—	—
**NPV (probability of no. of persons with mpox shedding MPXV when MPXV was not detected in wastewater)**					
<1	72.9 (70.5–75.2)	72.3 (69.8–74.6)	80.3 (76.2–84.0)	79.6 (75.5–83.3)	77.8 (75.4–80.0)
<5	92.3 (90.8–93.7)	92.4 (90.9–93.7)	94.6 (92.0–96.6)	95.1 (92.6–96.9)	94.9 (93.5–96.0)
<10	96.2 (95.0–97.1)	96.1 (94.9–97.0)	98.1 (96.3–99.2)	97.9 (96.0–99.0)	98.1 (97.2–98.8)
<15	97.8 (96.9–98.5)	97.6 (96.6–98.3)	99.1 (97.6–99.7)	99.1 (97.6–99.7)	99.2 (98.5–99.6)
<20	98.7 (98.0–99.2)	98.6 (97.9–99.2)	—	—	—
<25	99.3 (98.7–99.7)	99.3 (98.7–99.7)	—	—	—
<30	99.6 (99.2–99.9)	99.7 (99.3–99.9)	—	—	—

**Abbreviations**: MPXV = *Monkeypox virus*; NPV = negative predictive value; PPV = positive predictive value.

* Probabilities for N persons with mpox shedding MPXV when MPXV was and was not detected in wastewater were also calculated.

^†^ Wastewater results were combined for all sites serving a county; if at least one site serving a county detected MPXV in wastewater on a given sample collection day, that day was considered a detection for that county, and otherwise a nondetection.

^§^ Persons with reported mpox were assumed to shed MPXV in their county of residence from the day of symptom onset until 25 days later. The number of persons with mpox shedding MPXV were summed to determine the number of persons with mpox shedding MPXV on each day in a given county.

^¶^ Wastewater results for a given sample collection day were compared with the minimum numbers of persons with mpox shedding virus on that day in a given county.

** Wastewater results were combined by calendar week; if at least one site serving a county detected MPXV in wastewater in a given sample collection week, that week was considered a detection for that county, and otherwise a nondetection. Wastewater results for a given sample collection week were compared with the average minimum numbers of persons with mpox shedding virus that week in a given county. Estimates for larger numbers of persons with mpox shedding virus are missing because cell counts when examining data by week were small.

^††^ The main analysis used the date of symptom onset (imputed and not imputed) for all calculations.

^§§^ This sensitivity analysis used the earliest date available for persons with mpox for all calculations. Imputed symptom onset dates were not used.

^¶¶^ This sensitivity analysis examined weekly results by a 7-day rolling average, rather than by calendar week. For each sample collection day, the sample collection day, 3 days before, and 3 days after was examined. If at least one site serving the county detected MPXV in wastewater during those 7 days, that sample was considered a detection, and otherwise, it was considered a nondetection. Wastewater results were compared with the average minimum numbers of persons with mpox shedding MPXV in the county during those 7 days.

### Positive Predictive Value

PPV for predicting the presence of at least one person shedding *Monkeypox* *virus* in a county on a given day or week was 72.6% (95% CI = 61.8%–81.8%) and 61.9% (95% CI = 48.8%–73.9%), respectively. When virus was detected in wastewater during a given week, the probability that five or more persons were shedding virus was 34.9% (95% CI = 23.3%–48.0%) and the probability that 15 or more persons were shedding virus was 20.6% (95% CI = 11.5%–32.7%).

### Negative Predictive Value

NPV for predicting the absence of any persons shedding *Monkeypox* *virus* in a county on a given day or week was 72.9% (95% CI = 70.5%–75.2%) and 80.3% (95% CI = 76.2%–84.0%), respectively. When virus was not detected in wastewater during a given week, the probability that fewer than five persons were shedding virus was 94.6% (95% CI = 92.0%–96.6%) and the probability that fewer than 15 persons were shedding virus was 99.1% (95% CI = 97.6%–99.7%).

### Additional Analyses

In sensitivity analyses examining varying shedding durations, weekly sensitivity and NPV decreased and PPV increased as shedding duration increased (Figure). Daily sensitivity, NPV, and PPV followed these same trends (Supplementary Figure 2, https://stacks.cdc.gov/view/cdc/140513). Results changed only slightly in other sensitivity analyses (Table 2).

**FIGURE:**
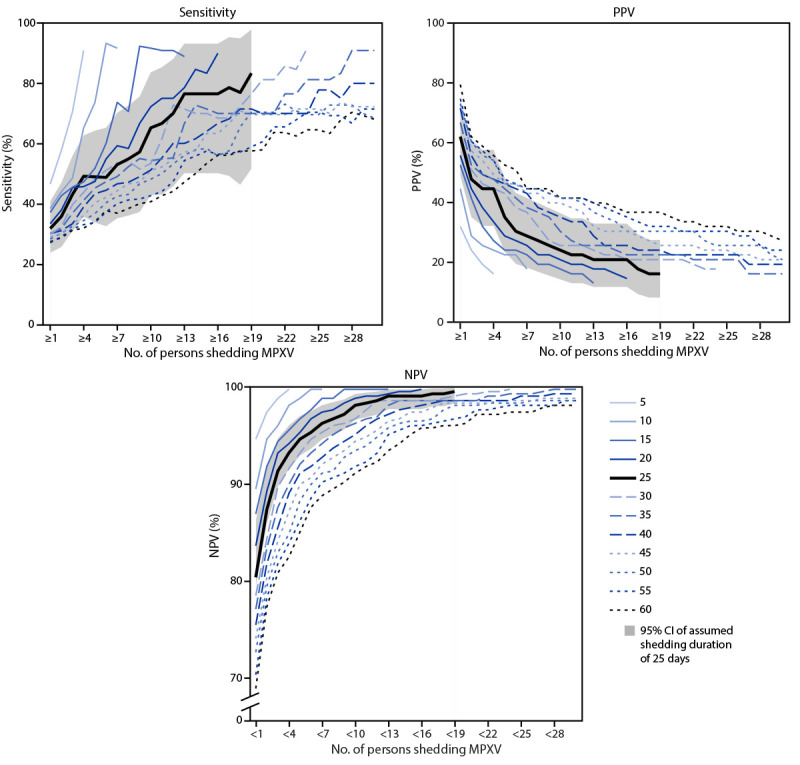
Sensitivity, positive predictive value, and negative predictive value* of wastewater surveillance^†^ for detecting persons shedding *Monkeypox virus*^§^ in a county in a week^¶^ for different assumed shedding durations** — United States, August 2022–May 2023 **Abbreviations:** MPXV = *Monkeypox virus*; NPV = negative predictive value; PPV = positive predictive value. * Sensitivity is the probability that MPXV was detected in wastewater when at least one person with mpox were shedding MPXV. PPV was defined as the probability that at least one person was shedding virus when a wastewater detection occurred. NPV was defined as the probability that no persons were shedding virus in the absence of wastewater detections. Probabilities for specific numbers of persons with mpox shedding MPXV when MPXV was and was not detected in wastewater are also shown. ^†^ Wastewater test results were combined for all sites serving a county: if at least one site serving a county detected MPXV in wastewater in a given sample collection week, that week was considered a detection for that county, and otherwise a nondetection. ^§^ Persons with reported mpox were assumed to shed MPXV in their county of residence from the day of symptom onset until 25 days later. The number of persons with mpox shedding MPXV were summed to determine the number of persons with mpox shedding MPXV on each day in a given county. ^¶^ Wastewater test results for a given sample collection week were compared to the average numbers of mpox cases shedding MPXV in that week in a given county. ** The assumed shedding duration was varied from 5 to 60 days in 5-day increments. Main results are shown with an assumed shedding duration of 25 days with 95% CIs. CIs were calculated using exact binomial tests.

## Discussion

This study is the first to examine the performance of wastewater surveillance for detecting mpox cases using empirical data. Wastewater surveillance had a sensitivity of 14% on a given day for detecting the presence of at least one mpox case. However, most sites were collecting more than one sample per week. Weekly sensitivity for detecting the presence of at least one mpox case was substantially higher (32%). As the number of cases shedding virus increased, weekly sensitivity increased to 49% for detecting five or more persons and 77% for detecting 15 or more persons shedding virus. Weekly PPV and NPV were both high (62% and 80%, respectively).

Although sensitivity might seem low compared with clinical testing, each wastewater sample represents thousands to millions of persons. Results show that wastewater surveillance was sufficiently sensitive to detect even a single mpox case in these large, pooled samples. These findings contrast those for SARS-CoV-2 (the virus that causes COVID-19), for which the minimum number of cases required for a wastewater detection is thought to be much higher (8–38 cases per 100,000 persons for detection rates at 50% and 99% probability, respectively) (*7*). Unlike SARS-CoV-2, high levels of *Monkeypox* *virus* are present in skin lesions as well as in urine and stool (*1**,**8**–**10*), and poxviruses are highly stable in the environment (*9*).

When *Monkeypox* *virus* was detected in wastewater on a single day or week, there was most likely (but not always) at least one case present in the county. Wastewater detections in the absence of known cases might have been the result of travelers, commuters, patients experiencing prolonged shedding, or subclinical or unreported infections (i.e., wastewater detections might have reflected true infections that had not been detected by case surveillance). Moreover, when *Monkeypox* *virus* was detected in wastewater on a single day or week, it was rare that 15 or more persons were shedding virus in the county. Because most samples were collected in fall 2022 or later, after case counts in the United States began to decline, large numbers of cases were infrequent. In addition, when *Monkeypox* *virus* was not detected in wastewater, there were most likely zero cases (and almost certainly no large numbers of cases) present in the county. High NPV can likely be partially attributed to low disease prevalence during the study period.

### Limitations

The findings in this report are subject to at least five limitations. First, because data on viral shedding patterns and clinical case information were lacking,***** variations in case shedding patterns were not included. Second, persons shedding *Monkeypox* *virus* might have resided within counties included in the analysis but outside areas covered by wastewater surveillance. This limitation would bias sensitivity and PPV estimates downward and NPV estimates upward, because wastewater surveillance cannot detect persons shedding virus if they reside outside covered areas. Third, because data from all sites serving a county were combined, results could not be stratified by sampling or testing methods or site population size and thus represent average estimates of wastewater surveillance performance across sites and time. Fourth, because case counts during the study period were low, estimates for detecting large numbers of cases are highly uncertain. Finally, although mpox is a nationally notifiable disease in the United States and all cases should be reported, and studies suggest that most cases are diagnosed, some cases might remain unreported (*6*).

### Implications for Public Health Practice

The findings in this report can help guide the public health response to *Monkeypox* *virus* wastewater detections. Because wastewater surveillance is sufficiently sensitive to detect very few mpox cases, a single, isolated wastewater detection might not warrant a large public health response. Moreover, because most wastewater detections during the study period resulted from five or fewer cases, the public health response to a single wastewater detection might be scaled to one recommended for small case numbers, as long as mpox case counts remain low. Finally, nondetection of *Monkeypox* *virus* in wastewater, in combination with no reported cases, can provide reassurance to public health officials that large numbers of cases are not present in communities where wastewater surveillance is occurring. Wastewater surveillance for *Monkeypox* *virus* has a sensitivity of 32% for detecting a single case, with sensitivity increasing to 49% and 77% for detecting five or more and 15 or more cases, respectively. PPV and NPV for *Monkeypox* *virus* wastewater surveillance are high (62% and 80%, respectively). Wastewater surveillance can be a useful complement to case surveillance for guiding the mpox outbreak response.

## Footnotes

*
https://www.cdc.gov/poxvirus/mpox/response/2022/index.html

†
https://www.cdc.gov/nwss/wastewater-surveillance.html

§
https://www.cdc.gov/poxvirus/mpox/cases-data/wastewater-surveillance.html

¶ NWSS’s commercial contractor began testing wastewater for *Monkeypox* *virus* in October 2022; it tested raw wastewater using quantitative polymerase chain reaction against a nonvariola Orthopoxvirus target that enables detection of both *Monkeypox* *virus* Clade I and Clade II. Detections were defined as samples with average concentrations >0 copies per liter. The academic partner program began testing wastewater for *Monkeypox* *virus* in July 2022; the program tested settled wastewater solid and primary sludge using droplet digital polymerase chain reaction. During July–December 2022, the program tested samples against a generic mpox target that enables detection of both *Monkeypox* *virus* Clade I and Clade II; a subset of samples was also tested using an assay specific to Clade II. In December 2022, generic mpox testing was discontinued, and all samples were tested with the Clade II assay. Detections were defined as samples with average concentrations >1,100 copies per gram.

** Jurisdictions reported case-level data to CDC electronically via a standardized case report form or the National Notifiable Disease Surveillance System (NNDSS). County of residence was reported for cases.

††
https://www.cdc.gov/poxvirus/mpox/clinicians/case-definition.html

§§ 2022 U.S. Census Bureau and 2021 State Health Department data (when U.S. Census Bureau estimates were not available by county) were used for county population estimates.

¶¶ Earliest county inclusion dates were the maximum of minimum sample collection dates for all county sites (minus 6 days to account for sampling on different days of the week). Last inclusion dates were the minimum of maximum sample collection dates for all county sites (plus 6 days) or May 7, 2023 (1 month before data download), whichever date was earliest. For duplicate sites (collecting samples for two sources), dates were included if at least one source was collecting samples.

*** Twenty-five days is the median time from illness onset to viral clearance in skin lesion samples, the sample type with the longest clearance time and highest viral load.

††† Other clinical dates included diagnosis date, date first reported to a public health department, first positive mpox test result date, rash onset date, case investigation start date, CDC case report date, and hospital admission date.

§§§ Median lag times from symptom onset date were calculated from the full case data set (30,026 cases). Negative lag times and implausible dates were excluded from calculations. In the study data set, symptom onset date was changed to rash onset date if rash onset was earlier (four cases). The earliest date available for most cases (60%) with missing symptom onset date (59 cases) was diagnosis date (median lag time = 6 days).

¶¶¶ Sensitivity = true positive (TP) / TP + false negative (FN); PPV = TP / TP + false positive (FP); NPV = true negative (TN) / TN + FN. TPs and FNs were defined as days or weeks with wastewater detections and no detections, respectively, and cases shedding. TNs and FPs were defined as days or weeks with no detections and detections, respectively, and no cases shedding. CIs were calculated using exact binomial tests. Specificity was not included because of limited utility in this context.

**** Equations for sensitivity, PPV, and NPV were used for calculations; however, the definitions of TPs, FNs, FPs, and FNs were changed. TPs and FNs were defined as days or weeks with wastewater detections and no detections, respectively, and at least a specified number of cases shedding. TNs and FPs were defined as days or weeks with no detections and detections, respectively, and less than a specified number of cases shedding. For example, FPs for detecting five or more cases were days or weeks with wastewater detections and fewer than five cases shedding.

†††† For each day in a given county, samples collected on that day and within 3 days earlier or later were included. If *Monkeypox virus* was detected in any of the samples, that day’s 7-day period was considered a detection, and otherwise it was considered a nondetection. Wastewater results were compared with the average number of cases shedding over the 7-day period.

§§§§ 45 C.F.R. part 46; 21 C.F.R. part 56; 42 U.S.C. Sect. 241(d), 5 U.S.C. Sect. 552a, 44 U.S.C. Sect. 3501 et seq.

¶¶¶¶ Cases were included in the analysis if they shed *Monkeypox virus* during dates of inclusion for county of residence; 281 cases were included in the main analysis (assuming 25 days of shedding). The number of cases included in all analyses ranged from 203 (assuming 5 days of shedding) to 632 (assuming 60 days of shedding).

***** Information on case characteristics, including immunosuppressing conditions and vaccination status, was frequently missing (≥60% of cases) or absent from the data set.
